# Lectures on Diseases of the Skin

**Published:** 1886-10

**Authors:** Henry Wile

**Affiliations:** Lecturer on Dermatology, Atlanta, Ga.


### LECTURES ON DISEASES OF THE SKIN.

DELIVERED AT THE ATLANTA MEDICAL COLLEGE DURING THE SESSION OF 1885–6, BY HENRY WILE, M. D., LECTURER ON DERMATOLOGY, ATLANTA, GA.

### LECTURE VIII.

CLASS I. DISORDERS OF SECRETION—HYPERIDROSIS—ANIDROSIS—BROMIDR—OSISOSMIDROSIS—CHROMIDROSIS—HEMATIDROSIS—URIDROSIS—SUDAMINA—SEBORRHŒA.

Reported Stenographically for the ATLANTA MEDICAL AND SURGICAL JOURNAL by W. Kay Tewksbury.

The affections embraced by this class are of such common occurrence that clear and positive ideas as to their pathology and therapeutics are essential in their successful treatment.

HYPERIDROSIS (excessive sweating) is that condition in which there is *primarily* an unusual and more or less chronic functional activity of the sweat glands, whereby the quantity of the secretion is increased. There are two forms called respectively *hyperidrosis universalis* and *hyperidrosis localis*.

Hyperidrosis universalis is met with in middle age or later years of life, also in childhood. When the sweating is profuse it gives rise to sudamina, especially in the delicate skin of the infant. It also exists secondarily as a symptom in acute fevers, rheumatism, tuberculosis and phthisis.

Hyperidrosis localis usually affects the face, scalp, genitals, axillæ, palms and soles—one or several of these localities, the last three being the favorite seats. It may appear on one side of the body as in disease of the vertebræ, or on one side of the head as in epilepsy and in hemicrania, or even in certain limited regions of the body as along particular nerve tracks. The disease is often a source of discomfort, even distress, and when affecting the palms of the hands it may incapacitate the individual for various kinds of special work. The palms have a cool, moist, clammy feeling, necessitating frequent drying and, under social circumstances, occasioning deep embarrassment. In the axilla, and about the genitals, where the surfaces are in apposition and there is not so much chance for evaporation, the perspiration is confined, staining the garments in the neighborhood, and, undergoing decomposition, acquires a peculiar odor. The same is true in regard to the plantar surfaces of the feet. The secretion is confined by the socks and shoes, causing more or less maceration of the epidermis, which leads to an exfoliation of the epidermal layers and formation of fissures. This renders the parts tender and painful.

The disorder may be of short duration or continue indefinitely. It is encountered in both sexes at all ages, but mostly in the young, and is generally associated with an anæmic, chlorotic or debilitated condition, though sometimes, but exceptionally, in apparently healthy individuals. As the action of the sudoriferous glands is under the influence of the vaso-motor nerves, the increased secretion must in great measure be due to some nerve disturbance, resulting in paralysis of the vaso-constrictors or stimulation of the vaso-dilators. Thus the affection has been observed in the course of many cerebral diseases, neuralgias of the cerebral nerves, spinal diseases and neuralgias of the spinal nerves. Relaxation of the cutaneous tissues may also exist as a cause.

The process is regarded as almost entirely functional and accompanied by little or no structural change. Virchow (Virch. Archiv. Bd. xiii. p, 288), examining sections of skin taken from the breast of a patient who, suffering during life from excessive night sweats, died of phthisis, found the cells lining the sweat glands fatty, degenerated and occasionally an atrophied condition of the gland.

The prognosis in universal hyperidrosis is as a rule not favorable, and the attention of the physician should always be directed to the cause of the trouble, the constitutional disturbance. In local hyperidrosis, though some cases prove obstinate, the treatment is highly satisfactory.

In the universal form the internal use of sulphate of atropia $\frac{1}{100}$ to $\frac{1}{30}$ of a grain is followed by relief, but this is only temporary. Likewise white agaric one to eight grains, extract of aconite root $\frac{1}{8}$ to $\frac{1}{2}$ grain. But for permanent good the general condition of the patient must be improved by means of tonics as iron, quinine, arsenic and attention to hygiene and diet. The local treatment may consist in the use of cooling lotions, such as equal parts of alcohol and rosewater, followed by a powder of starch, or equal parts of starch and oxide of zinc.

In local hyperidrosis one of the first injunctions to the patient is cleanliness. The milder forms, affecting the axillary, genital regions, palmar and plantar surfaces, usually yield to simple measures, such as the use of astringent washes and powders.

Tannic acid ten to thirty grains to a half pint of water; extract aconite ten grains to alcohol and ether each four ounces; a saturated solution of boracic acid; naphtol ten to forty grains to the ounce of deodorized alcohol, will all be found of value. Painting the parts with pure tincture of belladonna often acts happily, but it must be used with caution on account of its toxic effects. Bulkley recommends for most cases chloral, one ounce to the pint of water. These lotions should be applied freely for three to ten minutes at a time, two or three times a day, according to the severity of the case, each application being followed by the use of some absorbent powder. The following may be found to be of advantage: Salicylic acid ten grains to the ounce of starch; burnt alum one drachm to salicylic acid and Venetian talc, each two ounces. Where the skin is in apposition, as in the groins or between the toes, the powder should be used with pledgets of absorbent cotton. Sometimes a case will resist ordinary measures, and, being obstinate, will require persistent, systematic treatment.

In hyperidrosis of the soles, when the simple means indicated above fail to effect a cure, the diachylon ointment treatment, as originally recommended by Hebra, will give satisfaction. Diachylon ointment is composed of litharge and olive oil in the proportion of one part to four. They are mixed and allowed to boil slowly to the consistency of an ointment, after which oil of lavender is added in quantity one-tenth the amount of litharge. The ointment is spread on linen and applied to the soles of the feet, which should be previously washed and carefully dried. The plasters may be kept in position by means of a thin gauze bandage or socks. The application ought to be made twice daily for fourteen days, each time the surface of the skin being rubbed gently with dry, absorbent cotton. It is better for the patient to keep a recumbent position during the treatment, or walk as little as possible. Water is also to be kept from the parts during the treatment. The old and diseased epidermis gradually exfoliates, and a new, healthy surface is exposed. The use of some absorbent powder should be kept up a few days longer. Occasionally the procedure may have to be repeated, but by this means a thorough cure can be effected.

The internal treatment consists in the administration of tonics, where these are indicated for the correction of any debilitated condition of the system. For its direct inhibitory action upon the secretory apparatus of the skin, atropia in gradually increasing doses has been recommended.

ANIDROSIS is a rare condition, in which there is a diminished or arrested secretion of the sweat. It may be symptomatic of certain constitutional diseases, as cancer, diabetes mellitus and insipidus, tuberculosis, tabes dorsalis and often follows certain spinal diseases as myelitis and poliomyelitis, being regarded by some as a valuable differential symptom. It is also associated with a number of cutaneous diseases, as ichthyosis, psoriasis, xeroderma, chronic eczema, elephantiasis Arabum and prurigo, the function of the sweat glands occupying the diseased part of the skin being partially or wholly arrested. As in hyperidrosis it may be universal, or confined to a limited space, or along the track of a nerve, especially when the nerve has suffered mechanical injury. Relief depends upon the proper treatment of the primary disease of which it may be the symptom. Baths with friction are recommended.

Where profuse sweating continues, especially in parts that are confined, as on the feet or in the axillæ, and is neglected, there soon develops the condition known as *bromidrosis*. The secretion, more or less absorbed by the horny layers of the epidermis, and soaking into the clothing or leather of the shoes, undergoes chemical decomposition and emits a disagreeable odor. It has been shown (Neumann, Lehrbuch der Hautkrankheiten, 1880) that if the soles of the feet are kept scrupulously clean, the secretion of the sweat glands will have no odor. Where, therefore, the hyperidrosis is allowed to develop or continue without interference, the secretion may undergo decomposition and the patient is obliged to wash the parts several times daily, or go around enveloped in an odoriferous cloud. Thus it becomes a loathsome affection. As a rule it results from neglect, but cases have been reported where, in spite of scrupulous cleanliness, the sweat would at times possess a most disgusting odor.

Conditions in which the sweat emits an odor, but not foul, are known as *osmidrosis;* thus in acute fevers, scarlet fever, small-pox, rubeola, there is often a peculiar odor. Then, again, cases are on record in which the sweat had the odor of fruits and violets. The odor is due to the qualitative changes in the secretion, whereby certain volatile fatty acids (formic, butyric, caprilic acids) are developed.

The treatment is in general the same as that for hyperidrosis. A saturated solution of boracic acid is often of great service.

CHROMIDROSIS, in which the perspiration has a color, as blue, green, yellow or red, is a rare affection. A chemical examination of the sweat in such cases has revealed the presence of coloring matter as Prussian blue, indigo, indican, phosphoretted oxide of iron and bile pigment. It occurs mostly in females, on the eye-lids, forehead, chest, abdomen, hands and feet, and in males it is usually met with on the scrotum. The blue sweat is the most common form. The causes are not known. It is generally associated with general debility, and supposed to be due to nerve influence. The treatment should be tonic.

HEMATIDROSIS, or bloody sweat, is due to an extravasation of blood into the coil of the sweat gland. The sweat contains blood corpuscles which come from the rupture of fine capillary vessels. It occurs in individuals in whom there exists a tendency to hemorrhages, and hemorrhage in other organs of the body usually occur. The affection *per se* is insignificant.

URIDROSIS, urinous sweat, in which elements of the urine are present in the secretion, has been observed in patients suffering from affections of the kidneys. Cases have been reported in which crystals of urea and chloride of sodium were found on the skin of uræmic patients.

SUDAMINA is a disorder consisting of pin-point to pin-head sized vesicles that may appear in cases of hyperidrosis. The eruption appears suddenly, without any inflammatory symptoms, mostly on the trunks or anterior surfaces of the body. The lesions appear quickly in successive crops, exhibit no disposition to rupture and disappear by absorption, leaving small dry crusts. It is a common symptom in febrile diseases, occurs often on corpulent individuals, and especially those working under the direct rays of the summer sun or in heated places and transpiring freely; also on those with delicate skin, as infants who, on account of abundant clothing or summer heat, transpire profusely. The vesicles are in the superficial layers of the epidermis. They result from an hyperæmia of the papillæ and an over-stimulation of the sweat glands, whereby the secretion of the gland is forced between the epidermal layers. Microscopic examination shows a swollen condition of the cells lining the ducts of the glands.

The treatment consists in the use of cooling lotions containing alcohol, orange flower water, cologne water, etc., followed by a powder of starch, talc or carbonate of magnesium.

SEBORRHŒA is a functional disorder of the sebaceous glands. These glands secrete an oily, unctuous substance which finds its way into the hair follicles or to the surface of the skin, as already pointed out. There are two forms of the disease, according to the state of the secretion, it being more or less fluid or oily—*seborrhœa oleosa* and *seborrhœa sicca*. The former is characterized either by collections of yellowish, fatty crusts and scales, or by an oily layer upon the surface, the latter by layers of dry scales. Both forms may occur together. The form depends upon the locality, the former appearing mostly upon the non-hairy parts, the latter upon the hairy parts of the body. It may affect the entire surface (*seborrhœa universalis*), or what is most common, certain localities, it being named according to the locality.

On the scalp, *seborrhœa capitis*, it is most frequently encountered, and in the infant it occurs in the form of a yellowish or dirty brown friable deposit or layer. This deposit results from the continuation after birth of a process which during fœtal life is physiological, namely, when the entire surface of the skin is covered with sebaceous material (vernix caseosa). In the adult the disease (dandruff) manifests itself in the form of more or less thick layers of yellowish white, somewhat oily scales, which, when present in considerable quantity, mat the hair to the scalp, so that if the hair is gently straightened the layers are broken up and the sebaceous deposit will adhere to the hairs. This condition may also exist on hairy parts of the face. The disease may occur at any period of life—is met with mostfrequently in blondes, and is usually chronic. When it is neglectedthe hairbecomes dry and harsh, and may fall out to such an extent as to give rise to *alopecia* (baldness).

Affecting the face, *seborrhœa faciei*, it occurs, as a rule, in the form of *seborrhœa oleosa*, especially on the forehead, nose and on the chin. The surface presents an oily, glossy, shining appearance. On the nose the secretion may dry and form small yellowish crusts, which, when carefully lifted from the surface, will be seen to have on their lower side short projections, which fit into the ducts of the sebaceous glands. Occasionally, in elderly persons, the dry variety (*seborrhœa sicca*) is seen on the face, around the forehead, temporal regions and cheeks in the form of irregularly shaped and sized brownish or black, flattened, crusted lesions, giving the part an unwashed appearance.

*Seborrhœa genitalium* occurs in the male, especially around the glans penis and inner surface of the prepuce, where there exists any degree of phymosis; the secretion of sebaceous matter may accumulate, and undergoing decomposition prove a source of irritation, giving rise to balanitis and balano-posthitis. In the female it attacks the clitoris and labia minora.

*Seborrhœa corporis* is a rare variety, and is met with mostly on the anterior and posterior parts of the chest. On the anterior surface variously sized, irregularly shaped reddish spots are seen, and they are studded with loosely attached, yellowish, oily scales. On the back the appearance is similar, except that the clothing, by friction, keeps the lesions comparatively free from scales.

The causes of seborrhœa are numerous. It may be associated with gastric and intestinal derangements, anæmia, and in the female with the uterine and ovarian disorders. It occurs at any age, but usually at the time of puberty, and often follows febrile diseases.

It is essentially a catarrhal process, consisting of a hypersecretion, and accompanied by little or no structural change in the gland tissue. In the acini of the glands, outlets, hair follicles and on the surface of the epidermis varying quantities of the glandular secretion may be found. If this secretion be examined under the microscope, it will be seen to consist of epithelial cells in different stages of fatty degeneration and disintegration, variously sized oil globules, much molecular debris and foreign matter. The process is due to an excessive proliferation and rapid fatty degeneration of the epithelial cells lining the acini and outlets of the glands. The cells undergoing this degeneration first become granular and dotted with minute oil particles, which latter gradually coalesce as the cells disintegrate and finally appear together with the cellular débris upon the surface of the skin. When the oily constituents of the deposit predominate, the process is called *seborrhœa oleosa*, when the cellular *seborrhœa sicca* or *squamosa*.

The diagnosis of this disease may only present difficulty in aggravated cases. On the scalp it may be confounded with squamous eczema, psoriasis, tinea tonsurans and favus. From the first two it is distinguished by an absence of inflammation by its being more or less diffused over the whole surface. In eczema the scales are thin and scanty; in seborrhœa they are abundant and occur in layers; in psoriasis the scales are large, dry and pearly, and when removed reveal a red, often bleeding base; in seborrhœa the scales have an oily feel, and when removed reveal a pale, anæmic, non-infiltrated surface. From the last two the absence of a fungus is sufficient to establish a diagnosis. On the chest it may resemble tinea circinata, but here also a microscopic examination of the scales, showing no fungus, settles the matter. On the face it may be mistaken for lupus erythematosus, especially in the early stages, yet the former is more diffuse, while the latter is sharply defined with irregular borders. Then again, on removing the scales in the latter, the skin is seen to be red and infiltrated.

